# predictive model of nosocomial infection in patients with upper urinary tract stones after flexible ureterorenoscopy with laser lithotripsy: A retrospective study

**DOI:** 10.12669/pjms.40.3.8855

**Published:** 2024

**Authors:** Yanqiu Xu, Ping Xu

**Affiliations:** 1 Yanqiu Xu, Department of Urology, Suzhou Hospital of Integrated Traditional Chinese and Western Medicine, 39 Xiashatang, Suzhou, Jiangsu Province 215000, P.R. China; 2 Ping Xu, Department of Orthopedics, Suzhou Hospital of Integrated Traditional Chinese and Western Medicine, 39 Xiashatang, Suzhou, Jiangsu Province 215000, P.R. China

**Keywords:** Upper urinary tract stones, Flexible ureterorenoscopy with laser lithotripsy, nosocomial infection, Predictive model

## Abstract

**Objectives:**

To construct a predictive model of nosocomial infection in patients with upper urinary tract (UUT) stones after flexible ureterorenoscopy with laser lithotripsy (FURSLL).

**Methods:**

Medical records of 196 patients with UUT stones who underwent FURSLL in Suzhou Hospital of Integrated Traditional Chinese and Western Medicine from December 2019 to December 2022 were retrospectively analyzed. Patients were divided into infected group or uninfected group based on the presence of infection during postoperative hospitalization. Univariate and multivariate logistic regressions were used to identify risk factors of postoperative nosocomial infections. A nomogram prediction model was constructed using R software. The predictive ability of the model was assessed using the receiver operating characteristic (ROC) curve.

**Results:**

A total of 54 patients (27.6%) developed nosocomial infections after FURSLL. Logistic regression analysis showed that older age, diabetes, preoperative urinary system infection, ureteral stricture, hydronephrosis, double J-stent retention time, and stone diameter were risk factors of nosocomial infection. The nomogram model was constructed based on these risk factors. The ROC showed that the area under the curve (AUC) of the model was 0.930 (95% CI: 0.890-0.970), and the sensitivity and specificity were 92.6% and 81.7%, respectively, indicating that the prediction model was effective.

**Conclusions:**

Risk of nosocomial infection in patients with UUT stones after FURSLL is affected by older age, diabetes, preoperative urinary system infection, ureteral stenosis, hydronephrosis, double J-stent retention time, and stone diameter. The nomogram prediction model, constructed based on the above factors, has good predictive value.

## INTRODUCTION

Upper urinary tract (UUT) stones are a common condition with a prevalence of approximately 7% - 14% worldwide^1^,^2^ that is characterized by painful urination, hematuria, and bladder irritation.^3^ Delayed stone clearance can result in obstruction of the ureter and renal pelvis, adhesion and even mucosal cancer.^3^,^4^ Flexible ureterorenoscopy with laser lithotripsy (FURSLL) is a commonly used, minimally invasive treatment method for UUT stones with minimal postoperative complications.^5^,^6^ However, FURSLL requires water injection to keep the surgical field clear, which may result in excessive perfusion pressure. In addition, the presence of infection due to stone obstruction can increase the risk of postoperative nosocomial infection, leading to sepsis, septic shock, and increased surgical risk.^6^,^7^ Therefore, it is important to understand the risk factors associated with nosocomial infections in patients with UUT stones who have undergone FURSLL, to prevent these infections from occurring.^3^,^7^,^8^

Currently, there are no unified standardized guidelines for the clinical assessment of risk factors of nosocomial infection in patients with UUT stones after FURSLL.^9^,^10^ Moreover, there is a significant variability both in risk factors and individual characteristics between patients. Nomograms are widely used in different clinical settings to predict the probability of an event based on the risk variables.^11^-^13^ Therefore, the aim of this study was to construct a nomogram prediction model of nosocomial infection in patients with UUT stones after FURSLL to optimize patient management.

## METHODS

A total of 196 patients with UUT stones who received FURSLL treatment in Suzhou Hospital of Integrated Traditional Chinese and Western Medicine from December 2019 to December 2022 were retrospectively selected for this study. Patients were allocated into the infected group (n=54) or uninfected group (n=142) according to whether they contracted a postoperative nosocomial infection. Univariate and multivariate logistic regression analyses were used to identify the risk factors for postoperative nosocomial infection in all patients. A nomogram prediction model was established to predict the probability of nosocomial infection in patients with UUT stones after FURSLL.

### Inclusion criteria:

- Patients diagnosed with urinary tract stones and underwent FURSLL.^14^
- Age >18 years old.
- Complete medical record.

### Exclusion criteria:

- Other serious diseases, organ dysfunction, and malignant tumor comorbidities.
- A history of surgery or trauma six months before surgery.
- Uncontrolled pulmonary or intestinal infections before surgery.
- History of long-term use of hormone or immunosuppressive drugs.
- Anatomical abnormalities of the urinary system such as horseshoe kidney or ureter malformation.
- Women who are pregnant.

### Ethical Approval

The Medical Ethics Committee of Suzhou Hospital of Integrated Traditional Chinese and Western Medicine approved this study (No. 2023-003, Date: 2023-Aug).

### Observation indicators

Age, gender, and body mass index (BMI) of each patient were recorded. Basic disease status of each patient was assessed, with a focus on diabetes, hypertension, preoperative urinary system infection, ureter ectasis, and ureter stenosis. Additionally, the size of the stones, presence of hydronephrosis, operation time, and retention time of double J stent were recorded.

### Statistical analysis

SPSS22.0 and R software version 4.0.0. were used for the analysis. The measurement data that conformed to a normal distribution, were expressed as (*χ̅*±S), and the inter-group comparison was assessed by independent sample t-test. Non-normally distributed data were expressed as medians and interquartile intervals, and the Mann Whitney U test was used for inter-group comparison. Counting data were expressed as n (%), and a Chi-squared test was used for comparison between groups. Univariate logistic regression was used to analyze potential risk factors of postoperative nosocomial infections, and multivariate logistic regression was used to identify the independent risk factors. Based on the identified risk factors, the “rms” package in R software was used to construct a nomogram prediction model. Lastly, a ROC curve was established to analyze the predictive efficacy of the model. Statistical significance was set at P<0.05.

## RESULTS

A total of 196 patients were included in this study. Of them, 54 patients developed nosocomial infections after the surgery (infection group). Cystitis accounted for 27.8% (15/54) of infections, with pyelonephritis accounting for 16.7% (9/54), prostatitis accounting for 37.0% (20/54), and epididymitis accounting for 18.5% (10/54) infections, respectively. The remaining 142 patients did not experience a nosocomial infection (uninfected group). The results of the univariate analysis showed that the patients in the infected group were older than those in the uninfected group (P<0.05). The rate of complicated diabetes, preoperative urinary system infection, ureteral stricture and hydronephrosis was higher in patients in the infected group (P<0.05).

**Fig.1:**
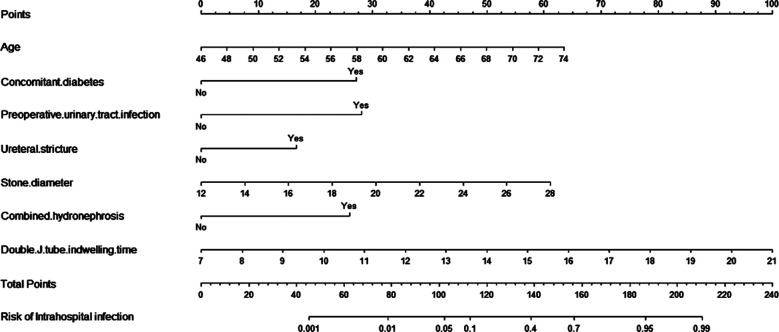
Nomogram prediction model.

The surgical time and double J stent retention time were longer in patients in the infected group (*P*<0.05). These patients also had larger stone diameter compared to the uninfected group (*P*<0.05) (Table-I). Multivariate logistic regression analysis showed that the risk factors for nosocomial infection after FURSLL were older age, diabetes, preoperative urinary system infection, ureteral stenosis, hydronephrosis, double J stent retention time, and stone diameter (Table-II). Based on the multivariate logistic regression model results, a nomogram prediction model was then created using the most significant risk factors, including age, concomitant dimensions, urinary system infection, ureal structure, stone diameter, combined hydrography, and double J stent retention time. The nomogram prediction model predicted postoperative nosocomial infections in patients and had a C-index of 0.930, suggesting good clinical efficacy (Fig.1).

**Table-I:** Comparison of clinical data between the two groups.

Index	Uninfected group (n=142)	Infected group (n=54)	χ^2^/t/z	P
Male [n (%)]	76(53.5)	26(48.1)	0.453	0.501
Age (years)	58.73±5.71	63.94±4.74	-5.973	<0.001
BMI (kg/m^2^)	25.77±2.25	25.84±2.12	-0.190	0.850
Concomitant diabetes [yes, n (%)]	9(6.3)	10(18.5)	6.630	0.010
Combined hypertension [yes, n (%)]	15(10.6)	8(14.8)	0.683	0.409
Preoperative urinary tract infection [yes, n (%)]	9(6.3)	14(25.9)	14.492	<0.001
Ureteral dilatation [yes, n (%)]	13(9.2)	7(13.0)	0.619	0.431
Ureteral stricture [yes, n (%)]	14(9.9)	16(29.6)	11.796	0.001
Stone diameter (mm)	18(15,20)	21(19,22)	-5.298	<0.001
Combined hydronephrosis [yes, n (%)]	17(12.0)	20(37.0)	16.050	<0.001
Operation time (min)	58(54,62)	63(58,67)	-4.219	<0.001
Double J stent retention time(day)	13(11,14)	16(14,17)	-6.347	<0.001

**Table-II:** Results of multivariate Logistic regression analysis.

Risk factors	B	SE	Waldx^2^	P	OR	95%CI
Age	0.147	0.048	9.509	0.002	1.158	1.055-1.272
Concomitant diabetes	1.730	0.777	4.954	0.026	5.642	1.229-25.890
Preoperative urinary system infection	1.911	0.776	6.065	0.014	6.757	1.477-30.910
Ureteral stricture	1.225	0.594	4.249	0.039	3.404	1.062-10.909
Stone diameter	0.246	0.085	8.357	0.004	1.279	1.082-1.511
Combined hydronephrosis	1.731	0.569	9.252	0.002	5.648	1.851-17.235
Operation time	0.061	0.041	2.198	0.138	1.063	0.981-1.152
Double J stent retention time	0.501	0.119	17.81	0.000	1.651	1.308-2.084

**Fig.2:**
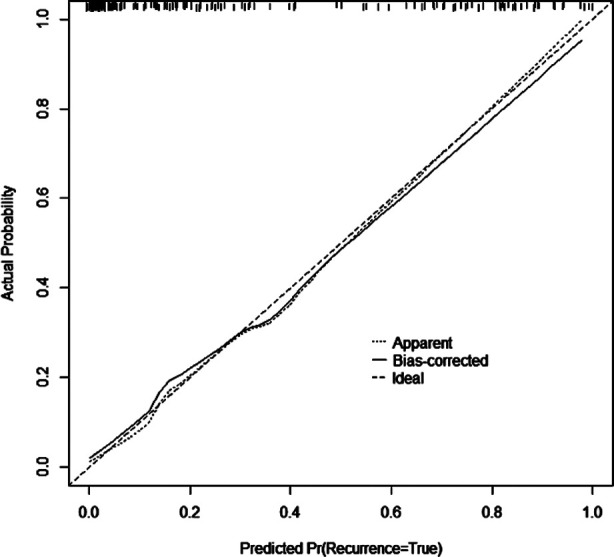
Predicted probability.

**Fig.3:**
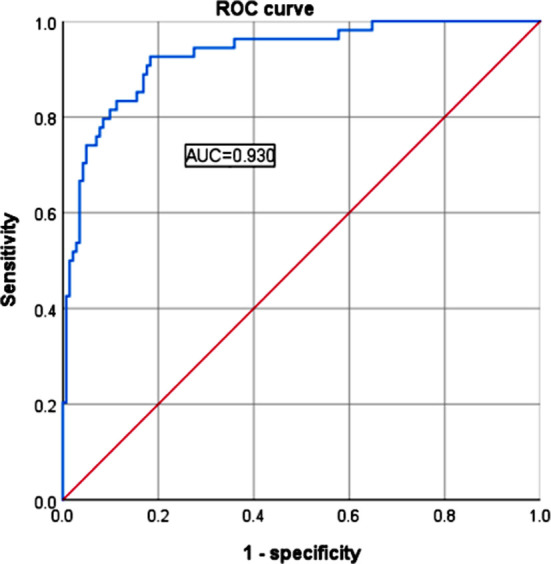
Receiver operating characteristics (ROC) curve of the nomogram prediction model.

The calibration curve also showed consistency between observations and nomogram predictions (Fig.2). The ROC was established to analyze the predictive capacity of the model in foreseeing postoperative nosocomial infection. The ROC AUC of the model was 0.930 (95% CI: 0.890-0.970), which suggests a certain predictive value (Fig.3). When the optimal cut off value was selected, the sensitivity and specificity were 92.6% and 81.7%, respectively, indicating that the predictive model is effective.

## DISCUSSION

Our study found that the rate of nosocomial infection after FURSLL for UUT stones is affected by older age, diabetes, preoperative urinary system infection, ureteral stenosis, hydronephrosis, double J stent retention time, and stone diameter. The nomogram prediction model constructed based on the above risk factors showed a good predictive value in patients with UUT stones who underwent FURSLL.

Postoperative nosocomial infections occurred in 27.6% of the patients in this study, which is higher when compared to the previous literature. Yuan et al.^15^ retrospectively collected data of 293 patients who underwent laser ureteroscopic lithotripsy, and found that 17.75% of patients developed postoperative infections, while Mi et al.^16^ reported that the incidence of infection after flexible ureteroscopy combined with holmium laser lithotripsy was 9.7%. The observed higher incidence in our results may be related to the smaller sample size and selection bias in this study.

Logistic regression analysis showed that the risk factors for nosocomial infection after FURSLL were older age, diabetes, preoperative urinary system infection, ureteral stenosis, hydronephrosis, double J stent retention time, and large stone diameter. Older age is associated with reduced physical and immune function and various chronic disease complications. As a result, older adults are more sensitive to anesthesia, surgical trauma, require longer recovery time after surgery, and can be more susceptible to gram negative bacterial infections, increasing the risk of nosocomial infection.^17^ Patients with diabetes experience elevations in blood glucose that is excreted in urine, creating environment that is favorable for bacterial growth. High blood sugar can also reduce plasma osmotic pressure and increase the risk of infection.^18^ Moreover, patients with diabetes may have peripheral neuropathy, which can reduce circulation and oxygen supply to the lower body, cause bleeding disorders, and reduce the body’s ability to resist infection.^19^ Waseda et al.^20^ showed that some patients with UUT stones also had a urinary system infection before surgery. FURSLL treatment can cause damage to surrounding tissues, and promote microbial invasion through the damaged mucosa. Additionally, ureteral stricture can lead to obstruction of the upper urinary tract and hydronephrosis. The urinary tract stones can adhere to the surrounding tissues. Discharge of the stone can directly damage the mucosa, causing stones containing infectious ingredients, such as trimagnesium phosphate ammonium phosphate, to invade the damaged area increasing the risk of nosocomial infection.^21^,^22^ Long term indwelling of a double J stent can stimulate the urethral mucosa as a foreign body, and infection is more likely if aseptic conditions are not maintained during the operation.^23^ Larger stone diameter is also a risk factor as they are more difficult to discharge, which increases the degree of urinary system obstruction, hydronephrosis, and subsequently, the risk of secondary infection.^24^ Additionally, operation time is also a risk factor for nosocomial infection after FURSLL, as longer operation times extend exposure time of the urinary system to bacteria. Long surgery times are also associated with greater surgical trauma, thus increasing the risk of urinary tract infection.^25^,^26^ While we detected differences in surgical time between the two groups of patients, further logistic regression analysis did not find that prolonged surgical time was a risk factor for nosocomial infection after FURSLL. This may be related to sample size selection bias, and further research is needed in this regard.

Nomogram prediction model that was created based on the identified independent risk factors, showed good prediction effect. Our model allows clinical healthcare workers to take timely measures to prevent and respond to the independent risk factors from multiple aspects and perspectives, in order to reduce the occurrence of postoperative infections, promote patient prognosis, and ensure patient health. Our nomogram model could be used as an auxiliary tool in clinical practice to help medical staff develop and optimize patient management protocols.

### Limitations

This was a retrospective study which analyzed only 196 patients. Therefore, further studies with larger sample size are needed for more robust conclusions. Additionally, there was no extended follow-up to understand long-term risks associated with nosocomial infection post FURSLL. It is also possible that there are additional risk factors which were not assessed within the scope of this study.

## CONCLUSION

Nosocomial infection after ureteroscopic holmium laser lithotripsy for UUT stones is affected by older age, diabetes, preoperative urinary system infection, ureteral stenosis, hydronephrosis, double J stent retention time, and stone diameter. We found that the nomogram prediction model constructed based on the above factors has a good predictive value.

### Authors’ Contributions

**YX:** Conceived and designed the study, involved in the writing of the manuscript and is responsible for the integrity of the study. **YX and PX:** Collected the data and performed the analysis. All authors have read and approved the final manuscript.
